# Response of African Elephants (*Loxodonta africana*) to Seasonal Changes in Rainfall

**DOI:** 10.1371/journal.pone.0108736

**Published:** 2014-10-09

**Authors:** Michael Garstang, Robert E. Davis, Keith Leggett, Oliver W. Frauenfeld, Steven Greco, Edward Zipser, Michael Peterson

**Affiliations:** 1 University of Virginia, Department of Environmental Sciences, Charlottesville, Virginia, United States of America; 2 Fowlers Gap Arid Zone Research Station, School of Biological, Earth and Environmental Sciences, University of New South Wales, Kensington, New South Wales, Australia; 3 Texas A&M University, Department of Geography, College Station, Texas, United States of America; 4 Simpson Weather Associates, Inc., Charlottesville, Virginia, United States of America; 5 University of Utah, Atmospheric Sciences Department, Salt Lake City, Utah, United States of America; Queen Mary, University of London, United Kingdom

## Abstract

The factors that trigger sudden, seasonal movements of elephants are uncertain. We hypothesized that savannah elephant movements at the end of the dry season may be a response to their detection of distant thunderstorms. Nine elephants carrying Global Positioning System (GPS) receivers were tracked over seven years in the extremely dry and rugged region of northwestern Namibia. The transition date from dry to wet season conditions was determined annually from surface- and satellite-derived rainfall. The distance, location, and timing of rain events relative to the elephants were determined using the Tropical Rainfall Measurement Mission (TRMM) satellite precipitation observations. Behavioral Change Point Analysis (BCPA) was applied to four of these seven years demonstrating a response in movement of these elephants to intra- and inter-seasonal occurrences of rainfall. Statistically significant changes in movement were found prior to or near the time of onset of the wet season and before the occurrence of wet episodes within the dry season, although the characteristics of the movement changes are not consistent between elephants and years. Elephants in overlapping ranges, but following separate tracks, exhibited statistically valid non-random near-simultaneous changes in movements when rainfall was occurring more than 100 km from their location. While the environmental trigger that causes these excursions remains uncertain, rain-system generated infrasound, which can travel such distances and be detected by elephants, is a possible trigger for such changes in movement.

## Introduction

Conservation of elephant populations, particularly in areas where poaching has been prevalent, is a pressing ecological issue. The management of elephant populations in both protected and unprotected areas requires an understanding of the predominant historical movement patterns of family groups [1]–[2]. Beyond the mapping of past elephant movements, it is important to understand what environmental cues might trigger the movement of elephants from one area to another. In dry areas, one such environmental trigger could be rainfall, particularly when it occurs at the end of a prolonged dry season. It is likely that any change in movement from dry to wet season conditions—ranging from distant migration-like excursions to localized movement—may be influenced by the habitat of the region (water, vegetation, and terrain) and dictated by topographically confined seasonal food and water sources [3]–[4].

The relationship between elephants and rainfall is embedded in the mythology and legends of people living for centuries in close contact with these animals. Turkana legend in northern Kenya holds that sighting an elephant at the end of the dry season is a sign that rain is imminent [1]. The Samburu people, further south in Kenya, have a similar belief, where the sudden appearance of an elephant, after months of no rain, signals the coming of the rains [1]. In India, the elephant is believed to bring the monsoon rain and is considered to be allied to cumulus clouds [1].

Surprisingly little research has been conducted on how elephants that populate arid environments might respond to rainfall triggers. Lindeque and Lindeque [5] reported a response of elephants in the eastern end of the Etosha National Park and Damaraland (Namibia) to rainfall well removed from where the herds were and well in advance of the rains. Leggett [6] observed elephants in the Kunene region of Namibia changing their movements within 24 hours of distant rainfall, heralding the start of the wet season. Neither study provided a possible cause for the observed change in elephant movement, however.

Loarie et al. [7] attempted to determine whether there was an underlying order in the effects imposed by climate, water and vegetation upon elephant movement. They examined these relationships over an extended transect from Namibia to Mozambique (2500 km) over multiple years (2000–2006). Tracking was not conducted simultaneously over the entire transect but in separate areas for different years. They found that elephants moved consistently over greater distances in the wet than in the dry season and covered larger areas in the drier regions than in the wetter regions. Although the authors partitioned the year into dry and wet seasons for each of the twelve regions across this extensive transect, no details were provided as to the criteria used for this dry to wet partition.

In the study most closely related to ours, Birkett et al. [8], using tracking data from Global Positioning System (GPS) collared elephants over a 3-year period in the Kruger National Park (South Africa), determined major seasonal break points in the movement of these elephants. After identifying the week (within each year) when elephant movements changed significantly, they related the weekly mean rainfall from rain gages located within the ranges of those animals. The authors found that 56% of the tracked elephants increased their step length after the rainfall break point. However, they also identified significant interannual variability in the rainfall/movement relationships.

Kelley and Garstang [9], using observations from a system similar to the International Monitoring System for the detection of nuclear explosions, demonstrated that sound pressure levels generated by thunderstorms could be detected by elephants at distances greater than 100 km. They further speculated that, in the presence of a near surface (100 m) nocturnal inversion, elephants might be capable of locating the source of the sound.

The goals of this study were to determine whether elephants changed their movement behavior with changes in the rainfall regime and whether the changes in movement were in response to some specific remotely generated signal. Drawing upon the conclusions of Kelly and Garstang [9], we postulated that such behavior would be most clearly demonstrated in environments with protracted dry seasons broken by an abrupt and distinct change to wet conditions such as that experienced by the Kunene elephants of northwestern Namibia. We defined rainfall criteria that identified the transition date in each year from dry to wet conditions. We then examined movements of individually collared elephants relative to these rain events.

A record of continuous tracking of elephants over a period of years is required to provide multiple seasonal changes and potential movement responses. Here, we utilized an unusually long data set of GPS positions of 14 Namibian elephants observed from 2002–2009. This represents one of the most extensive records of elephant movements available and forms the foundation for this study. Owing to the extremely dry climate and rainfall seasonality of western Namibia, we used the rather abrupt transition from dry to wet conditions to determine whether a statistically significant change in elephant movement could be detected. The characteristic convective nature of the first significant rainstorms of the wet season allowed us to postulate that infrasound produced by these storms might represent a remotely generated signal of the onset of the rains that is detected and responded to by these elephants. (Note: In the sections that follow, data are used over time periods that range over four to eight years, the shorter data sets not being independent of the longer sets.)

## Data and Methods

The raw data for this study included GPS observations of Namibian elephants fitted with collars that transmitted GPS signals over extended time periods. These GPS signals, which were often available daily and which in some instances, maintained signal strength for five years, served as the dependent variable in our study. We related elephant movements to rainfall observations recorded by both surface rain gages and satellite retrievals from which surface rainfall amounts were inferred. Daily rainfall measurements were used to characterize the time of transition from the dry to the wet season as well as to identify wet periods within the dry season.

### Elephant GPS data

**Figure 1:**
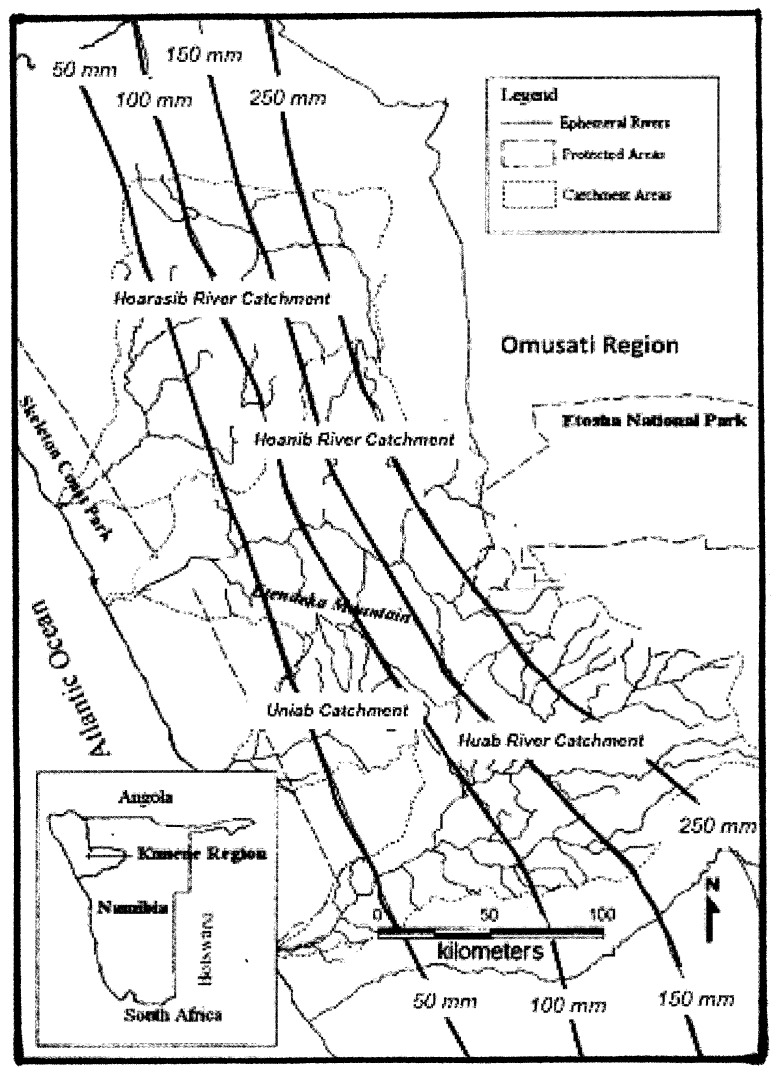
Western Namibia, showing the Hoarusib and Hoanib catchment areas of the western Kunene, and the Uniab and Huab catchment areas of the eastern Kunene together with the location of the Omusati region. Isolines (mm) of mean annual rainfall are superimposed [6].

The Kunene population of elephants in Namibia was essentially destroyed by poaching and military action in the Namibian War of Independence (1966–1989) and the Angolan War (1975–2002) [10]. By 2003, however, the total population in the western Kunene Hoarusib and Hoanib catchments (Figure 1) had rebounded to between 600 and 800 elephants [6].

Much less is known about the elephant population of the eastern Kunene Huab catchment region (Figure 1). Viljoen [11] estimated the population in the eastern Kunene to be about 180 in 1980. Poaching reduced this number by at least 60 by 1988. Like the western Kunene elephants, group sizes in the eastern Kunene are small, averaging around 3.82 in 2000 [12].

In September 2002, fourteen elephants were fitted with GPS collars [6]. In September 2004, the GPS units including batteries were updated on these fourteen elephants [10], [13]. These units functioned with mixed results until April 2009 over a total period of eight years. All eight years of tracking data was examined. However, of the 14 collared elephants, nine individuals had the longest continuous tracking record over a five year period from 2002 to 2006. This tracking record for these nine elephants formed the basis for the statistical analysis performed in this study. As noted below, when combined with the rainfall analysis to determine the transition from dry to wet conditions, the usable tracking data for the Behavioral Change Point Analysis (BCPA) lost another year (2002) of tracking data.

Of these nine elephants, three were located in the western Kunene (one male and two females) and five were in the eastern Kunene (four males and one female). The remaining elephant was a male from the western Omusati region (Figure 1) [12]. All males were sub-adults and assumed to be representative of their family units.

**Figure 2:**
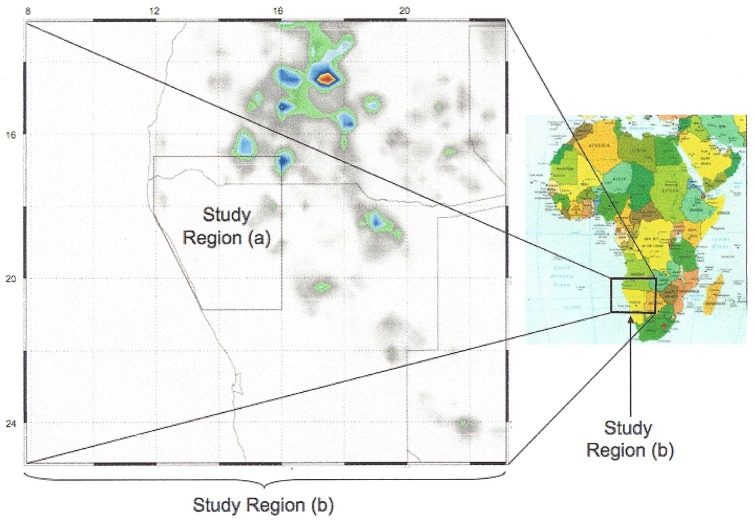
Rainfall study regions. (a) Surface and satellite-based rainfall study region, land areas from 12° to 16° east longitude by 17° to 21° south latitude (b) TRMM rainfall region—8° to 23° east longitude by 13° to 25° south latitude. TRMM located storms are shown for 30 December 2004.

Home ranges in Namibia are large, covering areas of 800 to 14000 km^2^, with Kunene elephants showing marked attachments to their home ranges [10], [14]. Females make only infrequent movements of greater than 20 km outside of the river channels [14]. Female ranges are limited by how far the young can travel, such that there is a tendency to stay close to natural permanent water [14]–[15]. Adult males tend not to be limited by water, drinking every three to five days, whereas females and family units drink every two to three days [14]–[15]. Rivers are ephemeral, sometimes not flowing in a given season or in successive years. For example, the Hoanib River flooded (i.e., contained running water) twice in the 2003 wet season for four days and one day respectively, and three times in the wet season of 2004 for seven, three, and four days [11]. Figure 1 shows the river catchments of the western and eastern Kunene regions of Namibia enclosed within the Study Region (see Figure 2a). Elephants routinely dig for water in river channels and will remain permanently in reserve areas, avoiding human populations [14]–[15].

The consideration of habitat including severe terrain, limited water and vegetation together with recent and extreme social dislocation may all influence the movement behaviour of these elephants. Such factors, while recognized, may go beyond the scope of this paper.

Permits from the Namibian Ministry of the Environment and Tourism (MET) were obtained for all of the years in which field work was conducted. Once MET permits are issued no other ethics permits are required by Namibia. Copies of these permits are available upon request. Archival tracking data collected by one of the authors (KL) is used in this study for these nine collared elephants over this five-year period. All relevant permits and permissions associated with the original collection of the data were complied with. No field work and no specific permissions were required in the re-analysis of these data presented in this paper. No elephants were harmed or injured in any way during the collaring or when the collars were removed.

### Rainfall

Rainfall estimates were made on two time and space scales. Figure 2 shows the space scale for the 4° longitude ×4° latitude “Study Region (a)” which includes the Kunene collared elephant range shown in Figure 1 and a much larger “Study Region (b)”, 15° longitude ×12° latitude, in which daily locations of storms are determined from the Tropical Rainfall Measurement Mission (TRMM) satellite and related to the collared elephants in Study Region (a).

Rainfall data in Study Region (a) were obtained from the National Oceanographic and Atmospheric Administration (NOAA) Climate Prediction Center's RFE20 [16]—a hybrid climatology of surface rain gage and satellite-based rainfall estimates not including the TRMM. Daily rainfall totals in Study Region (a) at 0.25°x0.25° or approximately 625 km^2^ spatial resolution were computed for the five-year period of elephant tracking (2002–2006).

**Figure 3:**
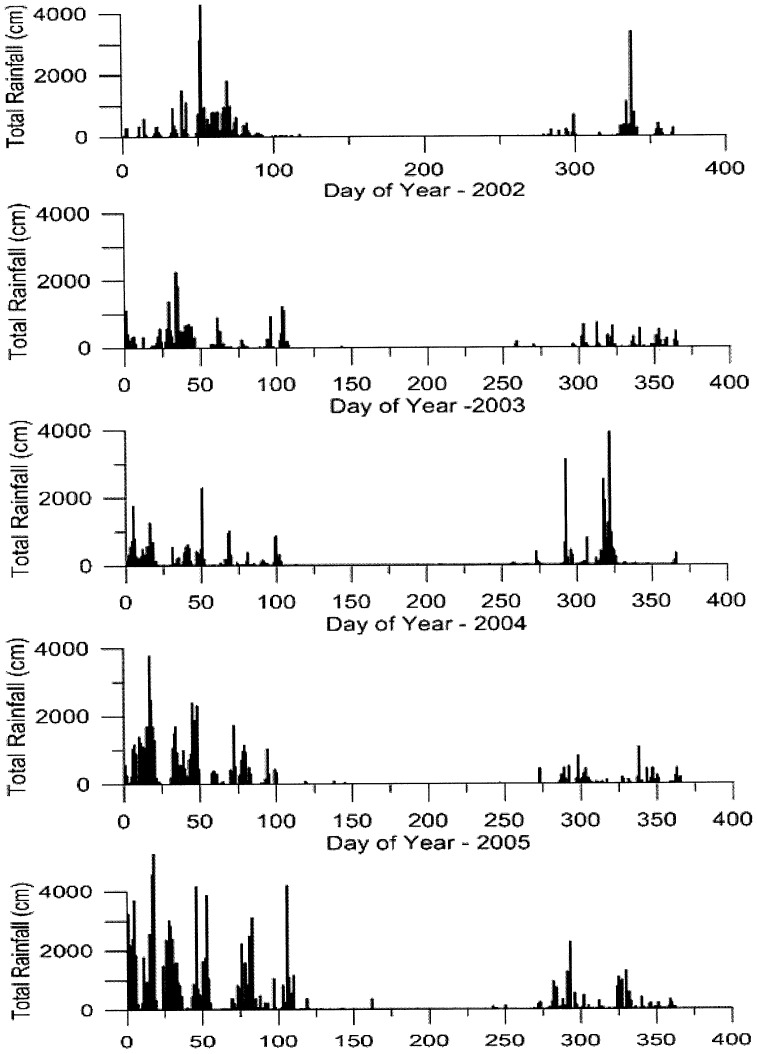
Daily total area rainfall is shown over the 400×400 km Study Region (a) for each of the five years (2002 to 2006) based on 0.25°x0.25° spatial resolution and determined from a hybrid climatology of surface raingage and satellite-based estimates of rainfall. The extreme rainfall of 6360 cm over the Study Region (a) on 21 February 2002 is only partially shown.

The daily rainfall summed over all grid elements in the 4°x4° Study Region (a) for each of these years is shown in Figure 3. Extremely dry conditions (no rainfall) are evident in consecutive months of most dry seasons. Rain episodes are observed in some dry seasons and those “within dry season” rain events are utilized in our analysis. The onset of the wet season is abrupt, beginning with low total rainfall, and in wet years, growing to higher total daily amounts as the wet season progresses.

The transition date from the dry to the wet season was determined for each year as the last day of a 24-day or more period during which no grid node in the 4°x4° Study Region (a) received more than 5 mm of rainfall in a given 24-hour period. This criterion ensures that dry conditions prevailed prior to the transition to wet conditions, and it increases the likelihood that a transition had in fact occurred and that it represented a distinct and abrupt change from dry-to-wet conditions. Choice of the magnitude (>5 mm of rainfall in 24 hours at any given grid point), and the preceding and succeeding number of dry and wet days (≥24 days), is based upon inspection of the five year rainfall record in the 4°×4° Study Region (a) shown in Figure 3.

Table 1 shows the Transition Date (TD) for each of the five years, the dates of the 24-day dry period preceding the TD, and the total amount of area rainfall recorded in Study Region (a) during the subsequent 24 wet-season days following the TD. Each year is then characterized as very dry (0–100 cm), dry (100–499 cm), moderately dry (500–999 cm), wet (1000–1499 cm), moderately wet (1500–1999 cm), or very wet (>2000 cm) where the rainfall represents the total amount of rainfall summed over all grid nodes of the 4°x4° Study Region (a).

**Table 1:** Dry season to wet season transition days based on the climatological record of rainfall in Study Region (a), see Figure 2 for detail.

Year	24 Dry Days		Transition Date day/month	24 Wet Days		Total Area (4×4 lat/long) Rainfall (cm)	Annual Rainfall Category
	Begin day/month	End day/month		Begin day/month	End day/month		
2002	12/09	05/10	05/10	05/10	28/10	1762.9	Moderately wet
2003	29/09	22/10	22/10	22/10	14/11	2312.9	Very wet
2004	07/09	01/10	01/10	01/10	25/10	4928.0	Very wet
2005	06/09	29/09	29/09	29/09	22/10	1635.8	Moderately wet
2006	06/08	29/08	29/08	29/08	21/09	334.7	Dry

In addition to recording the TD between the dry and wet seasons, we also identified wet periods within the dry season. As for the dry season, a 24-day dry period must precede the occurrence of rain with no grid element receiving more than 5 mm of rain in 24 h in Study Region (a). Table 2 shows the occurrence of such wet periods in three of the five years as well as the total amount of rain recorded in Study Region (a) for each wet episode (which in this case varies in length). The TDs fall between the end of August and the third week of October, an interannual variation of 54 days or approximately the two-month period of September and October.

**Table 2:** Wet periods within the dry season based on the climatological record of rainfall in Study Region (a), see Figure 2 for details.

Year	24 Dry Days		Transition Date day/month	Wet Days		Number of Days	Total Area Rainfall (cm) Category
	Begin day/month	End day/month		Begin day/month	End day/month		
2002	15/06	08/07	08/07	08/07	09/07	1	90.5
2003	22/08	14/09	14/09	14/09	28/09	15	402.0
2004	20/08	12/09	12/09	12/09	01/10	24	810.7

Examination of the eight years (2002–2009) of tracking showed gaps in data at critical times of the record (such as the transition from dry to wet conditions) reducing the usable record to essentially five years (2002–2006). Table 3 shows the details of the usable record for the nine remaining collared elephants that formed basis of our statistical analysis.

**Table 3:** Summary of GPS data availability for each elephant.

Elephant ID	Start Date	End Date	# GPS Fixes	Missing Data
EKM-02	1 Oct 2002	18 Jul 2006	1527	Apr-Sep 2004
				Jan–May 2005
WOM-01	6 Oct 2005	18 Apr 2007	327	
WKM-10	1 Oct 2002	11 Apr 2008	2017	Sep 2004
				Jan–May 2005
				May–Dec 2006
				Sep–Oct 2007
EKM-06	24 Sep 2004	4 Sep 2007	988	
EKM-03	1 Oct 2002	28 Jul 2007	1898	Jan–Aug 2004
EKF-01	1 Oct 2002	10 Apr 2004	1088	
WKF-16a1	1 Oct 2002	9 Dec 2003	967	
WKF-18	1 Oct 2002	2 Jan 2004	985	
EKM-01	1 Oct 2002	30 Nov 2006	288	Dec 2002–Sep 2004
				Dec 2004–Aug 2005
				Dec 2005–Aug 2006
EKM-07*	26 Oct 2007	30 Nov 2007	79	
WOM-06*	30 Oct 2007	20 Nov 2007	44	

Extended periods of missing data are listed in the last column. Tagged elephants are identified by a three-letter code followed by a number: E (eastern) or W (western); K (Kunene) or O (Omusati); and F (female) or M (male).

*although data are available for these elephants, the time periods are either too short or do not include enough data points prior to and after the main dry-wet transition period to permit analysis.

### Statistical analysis of the elephants' movements

The nine Namibian elephants tracked over the five years from 2002 to 2006 provided GPS fixes at varying time intervals, but typically no more frequently than once every 24 h. As the objective of this study was to determine whether significant changes in movement were related to pronounced changes in rainfall, locating the position of the GPS collared elephants at 24-hour intervals provided sufficient temporal resolution. In addition, the 24 h data collection interval maximized the length of the record by conserving battery power.

Individual movement patterns were examined using BCPA, a method previously developed for and applied to the study of migration patterns of large, marine mammals [17]. BCPA uses statistical likelihood analysis to identify structural shifts in animal movements—i.e., times when the animal's behaviour shifts between modes that could be identified by movement patterns, such as from foraging to migration. BCPA is designed to be robust to problems associated with data “gappiness” that are inherent in GPS tracking data in which observation times are often irregular. The BCPA method has been used in, or served as the basis for, movement analyses of birds [18]–[20] and marine mammals [17], [21], although to the best of our knowledge this approach has not previously been applied to elephant movements.

Specifically, an elephant's speed (V(T_i_)) is calculated as the simple absolute first derivative of movement between time steps,
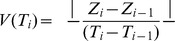
where Z_i_ is the position of the animal at time T_i_, with the index i-1 referencing the prior time step. The change in compass orientation over subsequent time steps is calculated as
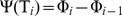
where 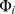 refers to absolute compass heading at time i. The tangential component of the elephant's movement vector is referred to as the persistence velocity (V_p_):
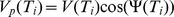



This variable tracks both the tendency for the animal's movement to be maintained in the current direction as well as the magnitude of that movement. Here we use the term “speed” to be consistent with the commonly used BCPA terminology—however, given that most of our GPS fixes are only available every 24 hours, the term “displacement” is probably more accurate. After calculating *V_p_* at each time step, with the BCPA method, a moving analytical window is passed through the time series and the mean, variance, and autocorrelation of *V_p_* are calculated both before and after the midpoint of the window. The size (length) of the window may vary. Thus, an increase in the mean of *V_p_* suggests that the animal's movement has become faster and more directed, an increase in variance indicates more stopping and starting, and an increase in autocorrelation implies more speed and directional persistence. BCPA identifies time periods when there are high densities of statistically significant changes in these statistical parameters. We then examine these potential behavioral “change points” in the context of background climate for that season. Complete details of the BCPA method can be found in Gurarie et al. [17].

Additionally, mean elephant movement rates were calculated over periods up to 24-days before and after the transition date from the dry-to-wet season as identified in the rainfall analysis. Rates were calculated from all available GPS fixes. An effort was made to balance the number of days (but not necessarily the number of rate calculations) before and after the transition date. In most cases, GPS locations were available at a 24-hour resolution. Mean differences were analyzed using a two-sample, two-tailed *t*-test. Levene's test for equality of variances was used to determine if data pooling was required in the calculation of the *t* test statistic.

## Results

### Statistical analysis of movement

**Figure 4:**
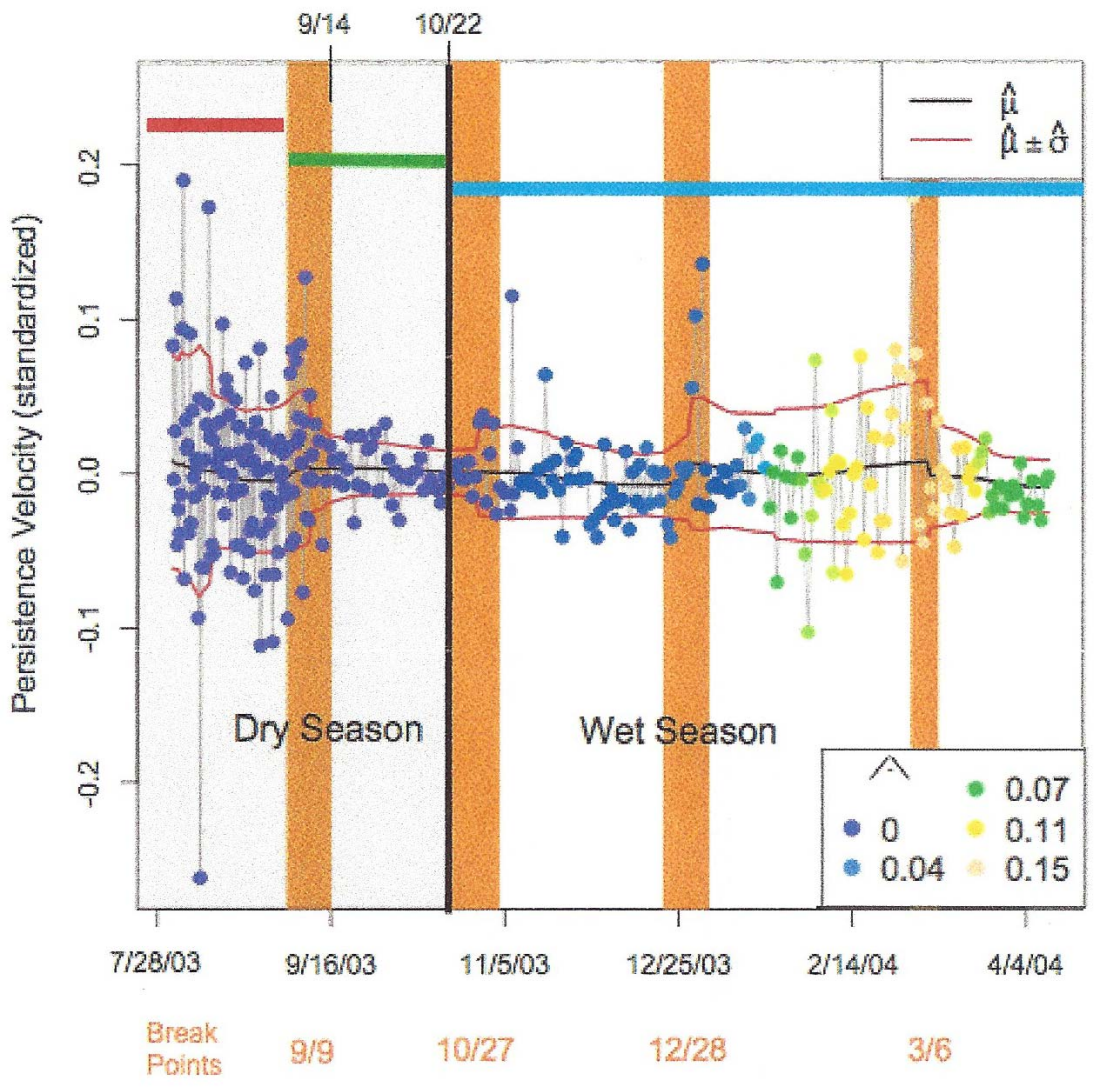
Behavioral Change Point Analysis (BCPA) results for elephant EKF-01 in 2003 showing the elephant's persistence velocity (V_p_). The mean, variance, and autocorrelation of V_p_ are tracked by passing a moving analytical window through the data. Times when statistically significant changes in these three parameters are observed are indicated by the vertical bars, and their midpoints are labeled below the horizontal axis (e.g., the first break point is 9/9). Colors of the circles are coded according to the autocorrelation of the persistence velocity, as shown in the key on the lower right. The dry–wet season transition occurred on 22 October and is indicated by the thick vertical line. Horizontal red and green bars show the 24 days before (red) and after (green) a within-dry season wet event; blue horizontal bar depicts the wet season.

BCPA results for *V_p_* for an Eastern Kunene female (EKF-01) are shown in Figure 4, where each point shows the relative tendency of the elephant to exhibit a persistent forward velocity. This is provided as an example of the output of the BCPA analysis for a single elephant tracked over one dry to wet season transition. During the 2003 dry season in August and early September, this elephant exhibited highly variable persistence velocities, as indicated by a range of *V_p_* values both well above and well below zero. A within-dry season wet event occurred on 14 September. Prior to this event, the elephant exhibited an increased persistence velocity and a decrease in *V_p_* variance, centered roughly on 9 September (see breakpoint 9/9 in Figure 4). Thus, there is evidence of a statistically significant movement change proximate with the timing of this rain event within the dry season. The main transition to the wet season in 2003 occurred on 22 October, and another behavioral change point is evident around 27 October. In this case, the variance of *V_p_* increased after the onset of the rain. This movement analysis for EKF-01 illustrates potential responses to multiple changes in rainfall including a pronounced change in movement occurring closest to a within dry season rain event.

**Figure 5:**
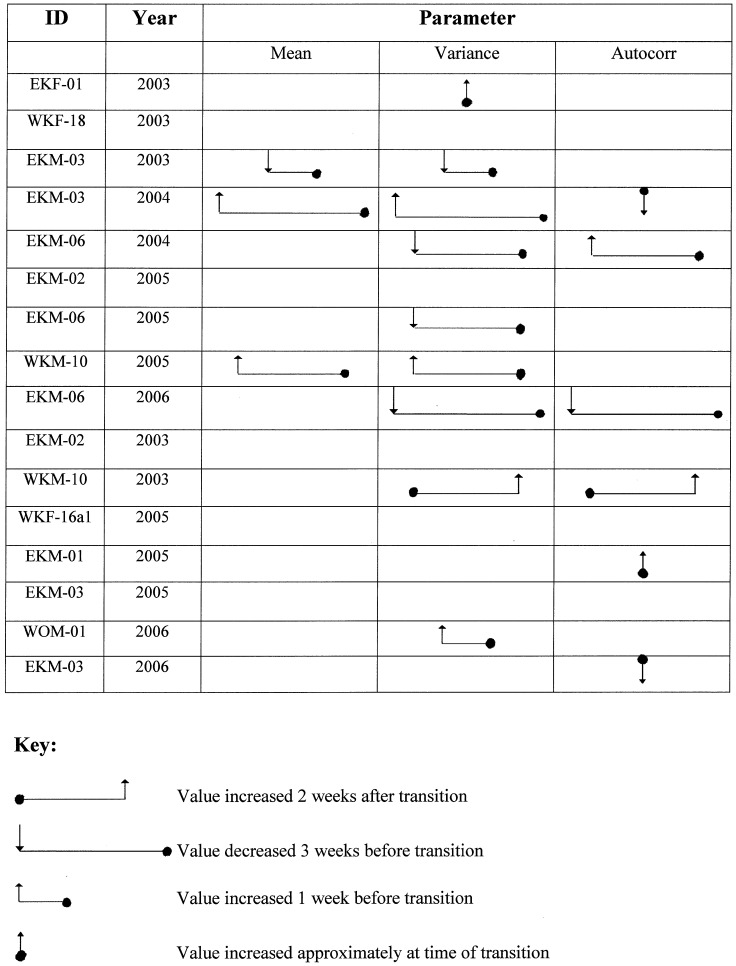
Summary of Behavioral Change Point Analysis (BCPA) of 9 collared elephants totaling 16 tracking years from 2003 to 2006 when BCPA indicated a significant change in the mean, variance, or autocorrelation of persistence velocity. Solid black dot is the dry-wet Transition Date (TD). Time on the horizontal axis increases to the right, i.e., movement changes occur after the TD and decreases to the left, i.e., movement changes occur before the TD; the horizontal axis, as shown in the key, is in increments of one week. Vertical arrow shows the direction of the parameter change: up  =  parameter increase; down  =  parameter decrease. Tagged elephants are identified by a three-letter code followed by a number: E (eastern) or W (western); K (Kunene) or O (Omusati); and F (female) or M (male).

A sequence of 15 BCPA results for the remaining elephant-years may be accessed at http://frauenfeld.tamu.edu/BCPA_Results/ for each of the nine elephants from 2003–2006. Tracks recorded in 2002, 2007, and 2008 were not subjected to this analysis because of a lack of GPS fixes recorded around the times of the dry–wet climate transition. As space does not allow for the complete presentation of all of these results, the BCPA movement change points and their relationship to seasonal rainfall transitions are summarized in Figure 5 for each elephant and season based on three weeks before and after the dry-to-wet rainfall TD. The statistically significant changes in the mean, variance, and autocorrelation of the persistent velocity presented in Figure 5 show that most changes in these quantities occur prior to the transition from dry to wet conditions. In seven cases, elephants showed a response prior to the transition, and three cases exhibited a response coincident with the change. Only one elephant responded following the occurrence of rain (WKM-10 in 2003). We conclude from these results that the tendency is for elephants to change their movement behavior before the onset of the wet season. Recall that the TD is determined for Region (a) (where the elephants are), whereas rains may have begun in some remote location (Region (b)), removed from where the elephants are. When the overall movement rate was examined for each elephant across seasons when GPS tracking data were available (Table 4), four of the nine animals exhibited statistically significant changes in rates of movement, with two elephants showing greater movement in the dry season than in the wet season, and two other elephants exhibiting the opposite trend. Three of the four animals were in the eastern Kunene region. Three of the elephants that did not exhibit significant speed changes were in the western area. All of the elephants that demonstrated significant movement changes were male, but our dataset only contains 3 collared females, so the limited sample size makes further inferences difficult.

**Table 4:** Comparison of mean elephant movement rates (km/hr) prior to and after the dry-to-wet season transition dates and results of unpaired, two-sample *t*-test based on the rates 24 days before and after each transition.

ELEPHANT ID	PRIOR RATE	POST RATE	P-VALUE	# TRANSITIONS
EKM-02	0.14	0.19	0.08*	4
WOM-01	0.11	0.24	0.05**	1
WKM-10	0.24	0.29	N/S	4
EKM-06	0.21	0.11	0.003**	3
EKM-03	0.25	0.17	0.01**	5
EKF-01	0.09	0.13	N/S	2
WKF-16a1	0.35	0.44	N/S	2
WKF-18	0.16	0.14	N/S	2
EKM-01	0.12	0.17	N/S	2

The number of seasons/transitions for which data were available for each elephant is shown in the last column.

** =  statistically significant difference at 0.05 level.

* =  difference at 0.10 level.

N/S  =  not significant.

**Figure 6:**
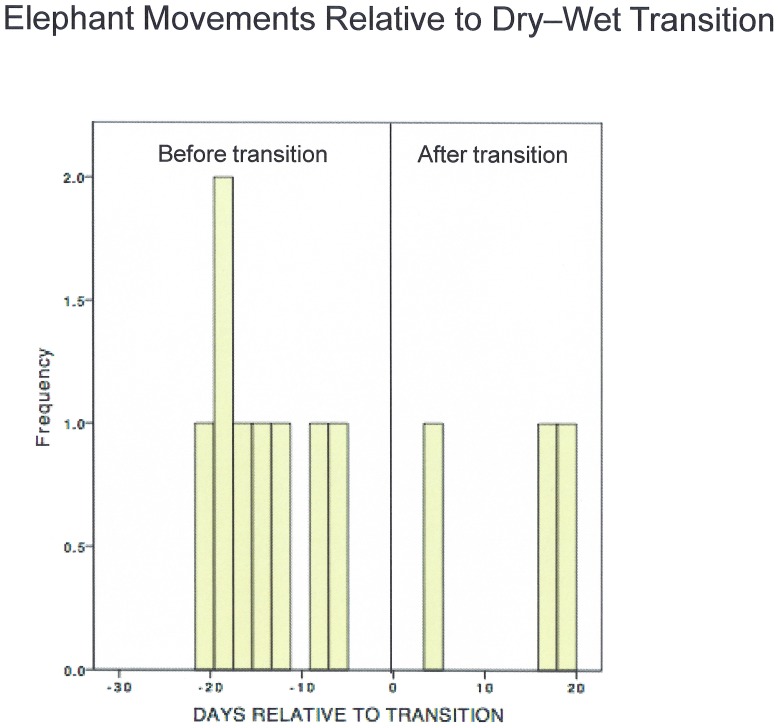
Statistically significant elephant movements in days before and after the transition date. Only changes observed within 24 days of the transition date are included.

To provide a summary comparison across all animals and years over the entire study period, we identified statistically significant movement periods (based on BCPA, see Figure 5 and Supplemental Materials) relative to the main TD for each elephant-year. Only movement changes that occurred within 24 days on either side of the TD are considered, and the middle day of any multi-day movement period is used in the calculation. More than twice as many significant movement changes were observed prior to the TD (Figure 6). However, the nature of the response is inconsistent between elephant-years (e.g., in some cases, the mean or variance of V_p_ increases prior to the TD whereas in others it decreases) and in the few available cases with tracking data for the same animal over several seasons, the responses were not consistent.

The BCPA results presented as an example in Figure 4 and summarized in Figure 5 are available in complete form in the Supplemental Materials at http://frauenfeld.tamu.edu/BCPA_Results/.

### Elephant movements in response to rain

Table 5 identifies cases of more than one elephant exhibiting a change in movement under the same climatic conditions (same year). Such near simultaneous change in movement occurred within six days in three cases, within nine days in two cases, and within 13 days in one case. To examine the likelihood that two or more elephants would exhibit movement changes within a period of x days, we calculated the probability (p) of at least one day overlapping in x-day periods over the n-day window used for each BCPA run as: 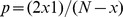



**Table 5:** Summary of near simultaneous change of movements of more than one elephant relative to the timing and location of the nearest rainfall event.

Year	Elephants	Change in Movement Dates month/day)	Probability	Nearest Rain Occurrence (month/day)	Rain Location (km)	Remarks
2003	EKF-01	27/10	0.15	22/10	280/ESE	Rain areas 100–300 km in E- quadrant from 02/10 to 08/11
	EKM-03	18/10		24/10	250/SE	
2003	WKM-10	07/11	0.119	08/11	100/E	Rain areas due E 100 km–400 km reaching SE corner of track region 08/11, 18 Z
	WKF-16a1	20/11		08/11	100/E	
2004	EKM-03	12/12	0.025	05/12	200/NE	Rain occurring from 07/11 to 07/12
	EKM-06	14/12		05/12		
2005	WKM-10	11/09	0.095	04/09	60 E/ENE from EKM-10	Weak rain systems 3 successive days
	EKM-06	17/09		04/09	75 NW from EKM-06	
2005	EKM-03	25/10	0.042	15/10–17/10	200 km E and SE	Rain (2.97 mm/hr) at edge of range on 16/10 at 18 Z
	EKM-06	28/10		15/10–17/10		
2006	EKM-06	11/08	0.149	31/08	Adjacent southern border of track region	Weak rain areas 05/08 to 30/08
	WOM-01	20/08		31/08		

Probability summarizes the likelihood that two (or three) elephants would exhibit significant movement changes within the number of days shown in column 3.

For example, in the first two rows of Table 5, x = 9 (there was a nine-day gap between the movement dates of different elephants in 2003) and n = 122 (the BCPA was run from 1 September through 31 December). Given these data and assuming randomness, the likelihood that two animals would undertake significant movement changes within six days of each other is 15% (Table 5).

**Figure 7:**
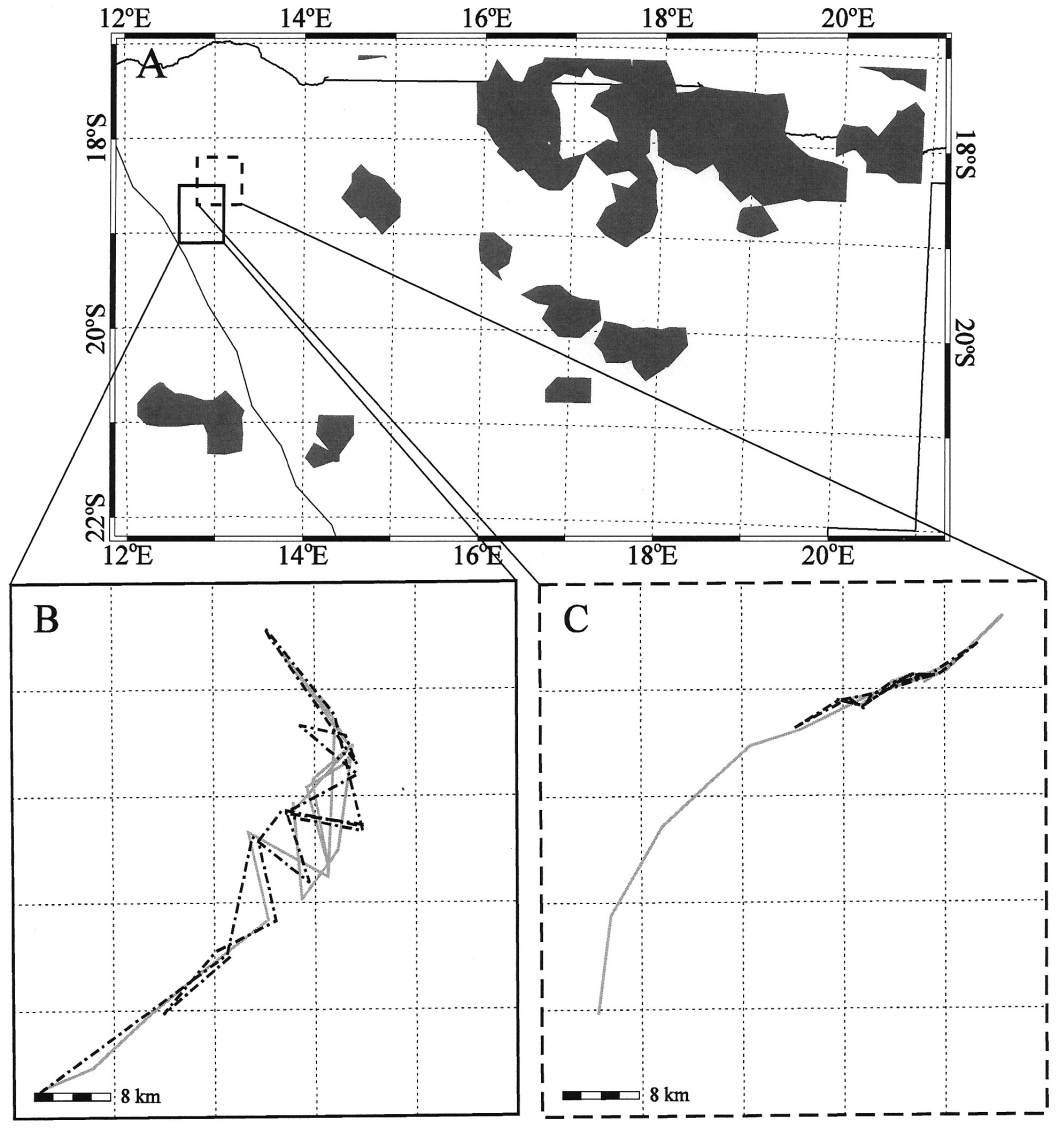
(a) TRMM 3-hourly rainfall distributions (grey) relative to the tracking rectangle for WKF-16a1 (solid rectangle) and WKM-10 (dashed rectangle) for 8 November 2003, with (b) tracks of elephant WKF-16a1 and (c) tracks of elephant WKM-10 for 2003 and 2004 (solid grey  =  wet season; dash-dot black  =  dry season; bar  = 8 km, see also Table 5).

**Figure 8:**
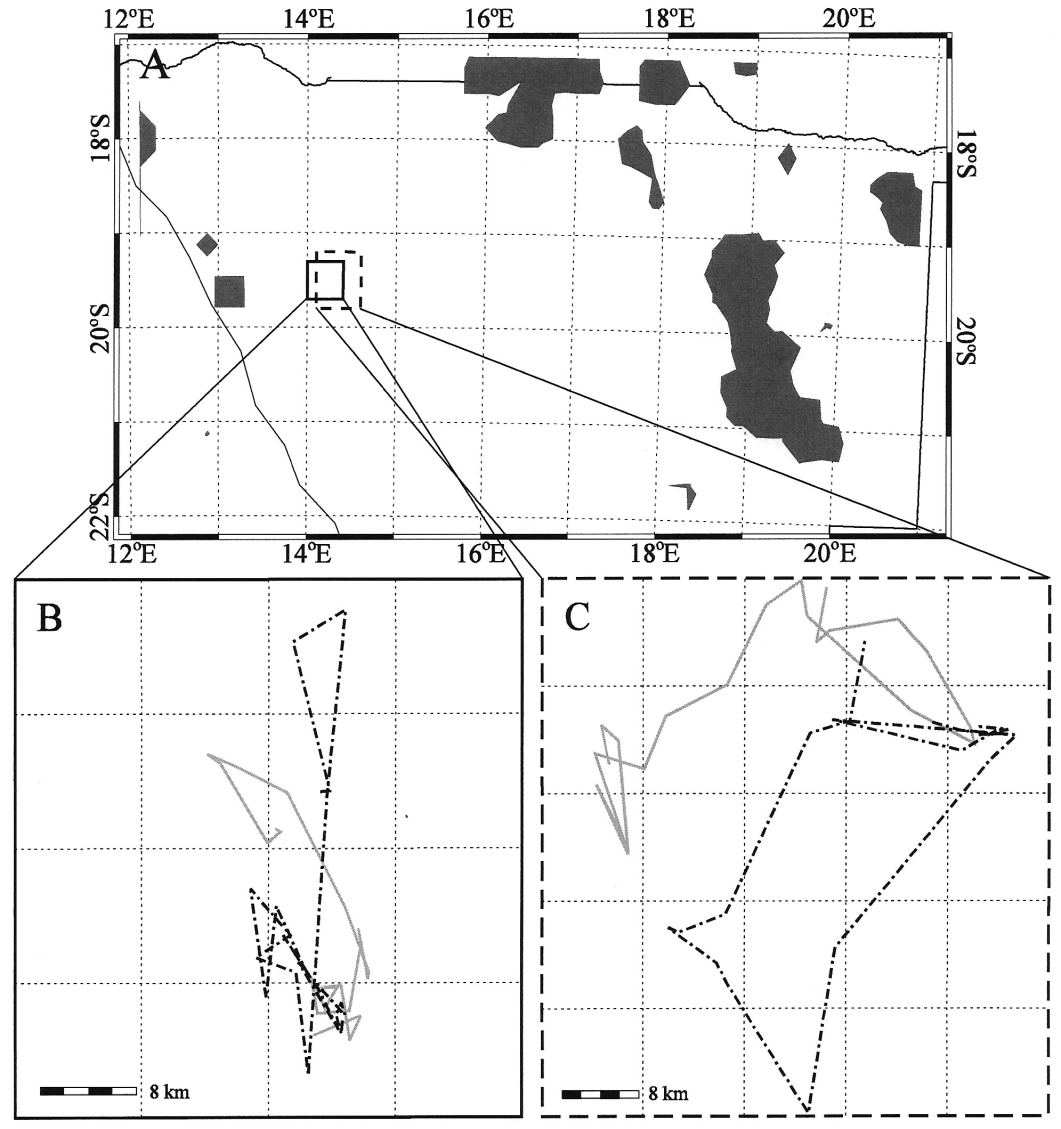
(a) TRMM 3-hourly rainfall distributions (grey) relative to the tracking rectangle for EKM-06 (solid rectangle) and EKM-03 (dashed rectangle) for 5 December 2004, with (b) tracks of elephant EKM-06 and (c) tracks of elephant EKM-03 for 2004 and 2005 (solid grey  =  wet season; dash-dot black  =  dry season; bar  = 8 km, see also Table 5).

In general, elephants changed their movements before any rain was observed at their location. Two elephants in different but proximal locations changed their movement behavior at or nearly at the same time on a statistically significant non-random basis in several instances. Rainfall was observed at distances between 65 and 280 km from their location (Table 5). In such near simultaneous movement cases, rain storms, as located by the TRMM satellite, occurred before or was approximately coincident with the change in movement in all but one case. In only one instance did rain follow the initiation of movement. We found no differences in movement or changes in movement between female and male elephants collared in this study. All males were sub-adults, suggesting that they were part of a family unit and that their movements were representative of the female-led family groups. The time between distant rain occurrence and the initiation of a movement change ranged from near coincidence to rain occurring of as much as 20 days after elephant movement. In the remaining four cases, elephant movement preceded rainfall by seven to 12 days, and the occurrence of rain was well removed (100–300 km) from the location of the elephants. Figures 7a and 8a show examples of the rain distributions and rectangles that enclose the movements of elephants WKM-10 and WKF-16a1 in 2003, and EKM-06 and EKM-03 in 2004. Figures 7b, 7c, 8b and 8c show the movements of the two pairs of elephants in 2003 and 2004. The regions that enclose the tracks of the elephants overlap but the tracks of each elephant are separate and distinct from each other. These rain distributions relative to the location of the elephants were used to determine the date, time, distance, and direction of the rain from the elephants documented in Table 5.

## Discussion and Conclusions

We conclude that a statistically significant change in movement was observed prior to or near the time of onset of the wet season. Such changes in movement also occurred prior to wet episodes within the dry season. In concert with results found by Loarie et al. [7] and Birkett et al. [8], changes in the movement of elephants were found to both precede and follow the transition from dry to wet conditions, with a preponderance of the statistically significant changes found in the 24 days prior to rather than in the 24 days following the dry–wet transition. In several instances, elephants in different (but proximal) locations exhibited near simultaneous change in movement, and these movements were unlikely to have occurred by random chance. This behavior suggests that the animals could be responding to a common environmental signal.

The transition from dry conditions to wet conditions was defined by a rainfall amount in a given grid element in a given area (Region (a)) preceded by 24 days with <5 mm/day in any grid element and followed by 24 “wet” days. This criterion provided a useful, objective method for identifying the transition date partitioning the dry from the wet seasons. A modified version of this criterion also served to identify wet periods within the dry season. Most significant wet season rain storms in this region are convective in nature and contain lightning. Lightning discharges may, however, occur in so called “dry storms” that produce little or no measurable rain [22]–[23]. These rain storms were located in a much larger region (Region (b)), potentially remote from the location of the elephants.

The above evidence suggests that elephants change their movement behavior both in response to a seasonal change in rainfall (dry to wet) and to wet episodes occurring in the dry season. The evidence presented suggests that such responses are triggered by rainfall occurring at some distant location, perhaps as much as 300 km from the location of the elephants. This, in turn, implies a signal coincident with, and perhaps produced by, the rain event itself [9]. Such a signal may be required to travel over distances greater than 300 km in time periods of much less than 24 hours [9]. Such distances are well within the threshold of detection of thunderstorms by elephants as determined by Kelley and Garstang [9].

The interaction of the elephants observed in this study with their habitat (terrain, vegetation and water) and with humans may have conditioned these elephants to remain in protected areas, frequent river channels, and limit their excursions outside of these known areas. Such conditioning may well play an important role in influencing the larger movement behavior of the current elephant population in northwest Namibia. No extended seasonal movements were observed in the Kunene region comparable to those reported by Lindeque and Lindeque [12] in Damaraland east of Etosha National Park.

Changes in vegetation may occur prior to the arrival of the rains and thus trigger movement before the rains arrive. Because such changes in vegetation are due to a complex set of factors including changing length of day, solar insolation, slope, aspect, soil type, and soil moisture, among others, it is less likely that this greening will trigger simultaneous changes in movement behavior by more than one elephant. Furthermore, such changes in vegetation do not occur in the dry season when we observe changes in movement by the elephants in response to rainfall. While it remains possible that smell generated by rain storms could produce a response, the transmission of olfactory signals detectable by elephants would depend both upon surface wind speeds and direction and upon concentrations [24]–[25].

We suggest instead that low-frequency sounds produced by rain storms [9] represent a more plausible explanation of a detectable signal generated by a distant rain event. Not all rainstorms may generate infrasonic signals and not all elephants will exhibit a response to such sounds. Nevertheless, the response documented in this study suggests that the elephants of western Namibia change their movement behavior roughly coincident with a change in the seasonal rainfall climate of the region. This change in movement occurs well before (days to weeks) any rain actually falls in the elephants' location. Although such a response is in near coincidence with the change from dry to wet season conditions, rain events within the dry season may also trigger a change in elephant movement. We hypothesize that such responses by elephants to spatially remote events could be ascribed to the detection of infrasonic signals generated by that event. The existing measurements, however, do not provide unequivocal evidence of this possibility. Given the temporal coarseness of the GPS measurements available and the lack of continuous, long-term monitoring for some animals, we realize that these limitations pose significant barriers to reaching definitive conclusions regarding the factors that motivate relatively sudden elephant movement changes. The lack of a consistent response (i.e., increasing vs. decreasing mean, variance, or autocorrelation) certainly adds additional complexity to our analysis. Additionally, we acknowledge that BCPA is but one method that can be used to retrospectively examine animal movements. A variety of methods are available (e.g., Fleming et al. [26], Yackulic et al. [27], Breed et al. [28], Boettiger et al. [29]), and we encourage their adaptation to problems allied with elephant tracking and conservation.

 While the elephants in the dry and rugged Kunene region of Namibia may change their movements in response to distant rainfall, these elephants do not leave their dry season range or head towards the early rainstorms. The learned and adaptive behavior of the Kunene elephants may not only reflect the exigencies of their habitat and climate but exhibit a residual response to the recent and extreme social dislocation suffered by this population. Additional study is clearly needed to understand what motivates these early seasonal changes in movement behavior.
